# The doctors of the future: the competition of ChatGPT-4, ChatGPT-4 omni, and Gemini 2.0 Flash in andrology

**DOI:** 10.1186/s12894-026-02248-5

**Published:** 2026-07-07

**Authors:** Umit Uysal, Ergun Alma, Adem Altunkol, Hakan Ercil, Mert Hamza Özbilen, Hakan Anıl, Kazım Yelsel, Burak Sağmak, Gökay Çetinkökü

**Affiliations:** 1 Department of Urology, Health Sciences University Adana City Training and Research Hospital, Adana, 01000 Turkey; 2 Department of Urology, Adana Yüreğir State Hospital, Adana, Turkey

**Keywords:** Artificial intelligence in healthcare, ChatGPT 4, ChatGPT 4o, Gemini 2 flash

## Abstract

**Objectives:**

Large language models (LLMs) are increasingly being used in medical research and as clinical decision-support tools. This study aimed to compare the accuracy and reliability of responses generated by large language models in response to andrology-related questions.

**Materials and methods:**

Seventy questions concerning diagnosis, treatment, and general information were developed on the basis of the 2024 Andrology Guidelines of the European Association of Urology (EAU). These questions were submitted to three large language models, namely ChatGPT-4, ChatGPT-4o, and Google Gemini 2.0 Flash. The responses were independently evaluated by three senior urologists using a four-point rating scale. A total score (TS) > 9 indicated a good response, 6 ≤ TS ≤ 9 indicated a moderate response, and TS < 6 indicated a poor response. In addition, the self-correction capabilities of the models were evaluated, and changes in response accuracy after re-evaluation were analyzed.

**Results:**

ChatGPT-4o achieved the highest total scores in the diagnosis and treatment categories (*p* < 0.001). Google Gemini 2.0 Flash generated the longest responses but demonstrated the lowest accuracy. ChatGPT-4o also showed the greatest improvement following the self-correction process (Cohen’s d = − 1.214, *p* < 0.01). Fleiss’ kappa coefficient values ranged from 0.61 to 0.80, indicating substantial interrater agreement among the urologists.

**Conclusion:**

ChatGPT-4o emerged as the most reliable model for andrology-related questions, providing responses that are were highly consistent with current clinical guidelines. The self-correction capabilities of the models improved response accuracy, suggesting that error-awareness mechanisms in large language models have the potential for further refinement. Nevertheless, expert supervision remains essential for the safe implementation of AI-assisted systems in clinical practice.

**Supplementary Information:**

The online version contains supplementary material available at https://doi.org/10.1186/s12894-026-02248-5.

## Introduction

Artificial intelligence (AI) refers to technologies capable of performing tasks such as problem-solving, learning, language interpretation, and planning. Over the past two decades, AI has gained increasing prominence across various domains, including medicine, where its significance continues to grow [1]. The advancement of AI presents new opportunities to enhance healthcare services and enable personalized patient care. Large language models (LLMs) leverage natural language processing (NLP) and deep learning (DL) techniques to analyze and understand complex linguistic structures [2]. Currently, models such as ChatGPT, Gemini Advanced, and Copilot are notable for their advanced text comprehension and generation capabilities, particularly in medical literature analysis and patient communication [3].

The chat generative pretrained transformer (ChatGPT), developed by OpenAI, is an LLM designed to engage in dynamic dialogue with users and provide effective responses to inquiries. It has gained significant recognition, particularly for its performance in the medical domain [4]. On May 13, 2024, OpenAI introduced ChatGPT-4o, marking a significant advancement. Compared with ChatGPT-4.0, ChatGPT-4o has enhanced ability to understand complex queries and improve contextual awareness, thereby generating more accurate responses [5]. In addition to ChatGPT, various LLMs with different architectures have been developed, including Gemini, developed by Google AI, which is capable of text generation, translation, and query answering [6].

Recently, the effectiveness of AI-based models in disseminating medical information and serving as clinical decision-support systems has been evaluated in various studies. In a study evaluating the accuracy of ChatGPT-4, ChatGPT-4o, and Gemini Advanced in electrocardiography (ECG) analysis, ChatGPT-4o showed promise, particularly for more challenging ECG questions. However, its performance in general assessments has remained inferior to that of human experts [7]. In another study evaluating the accuracy of ChatGPT-3.5, ChatGPT-4.0, ChatGPT-4o, and Gemini in diagnosing potentially malignant oral lesions, ChatGPT-4o showed better performance but did not reach the level of expert physicians [8].

The aim of this study was to evaluate and compare the performance of OpenAI’s ChatGPT-4 and ChatGPT-4o, and Google’s Gemini 2.0 Flash in answering andrology-related questions on the basis of the 2024 European Association of Urology (EAU) Guidelines. In this context, the accuracy, consistency, and guideline concordance of responses to structured, guideline-based andrology questions were examined to characterize the performance of these models in delivering guideline-based medical information.

## Materials and methods

On the basis of information supported by high levels of evidence and strong recommendations in the 2024 Guidelines of the European Association of Urology (EAU), we formulated 70 questions related to sexual and reproductive health (Supplementary Table 1). The questions were selected on the basis of their applicability to common clinical settings. To ensure standardization, minimize interpretive variability, and allow a fair comparison across different large language models, the questions were deliberately designed to be guideline-based, clear, and fully specified. This structured format was intentionally chosen to assess factual accuracy and adherence to clinical guidelines rather than higher-order clinical reasoning or decision-making under uncertainty. To better characterize the strengths and weaknesses of LLM chatbots, we categorized these questions under the headings of male hypogonadism, erectile dysfunction, ejaculation disorders, penile curvature, penile size abnormalities and dysmorphophobia, priapism, and male infertility.

Additionally, the questions were classified into three key domains: general knowledge, diagnosis, and treatment. Between February 13 and February 20, 2025, these questions were presented in English to two versions of ChatGPT, namely ChatGPT-4 and ChatGPT-4o (OpenAI, California, USA), and to Google Gemini 2.0 Flash (Google LLC, Alphabet Inc., California, USA). Each question was entered as a separate conversation, and the conversation history was reset after each query to ensure unbiased data collection. This approach was adopted to ensure standardized and controlled comparisons across models and represented an idealized testing condition. The responses were then converted into plain text, with all information identifying the LLM chatbot removed, and were subsequently evaluated by three experienced urologists. These models were selected because they represented widely used and highly accessible large language model platforms in medical and scientific contexts at the time the study was conducted. All the models were evaluated under identical conditions and in the same language to ensure methodological consistency and comparability. The overall study design is illustrated in Fig. 1.

**Fig. 1:**
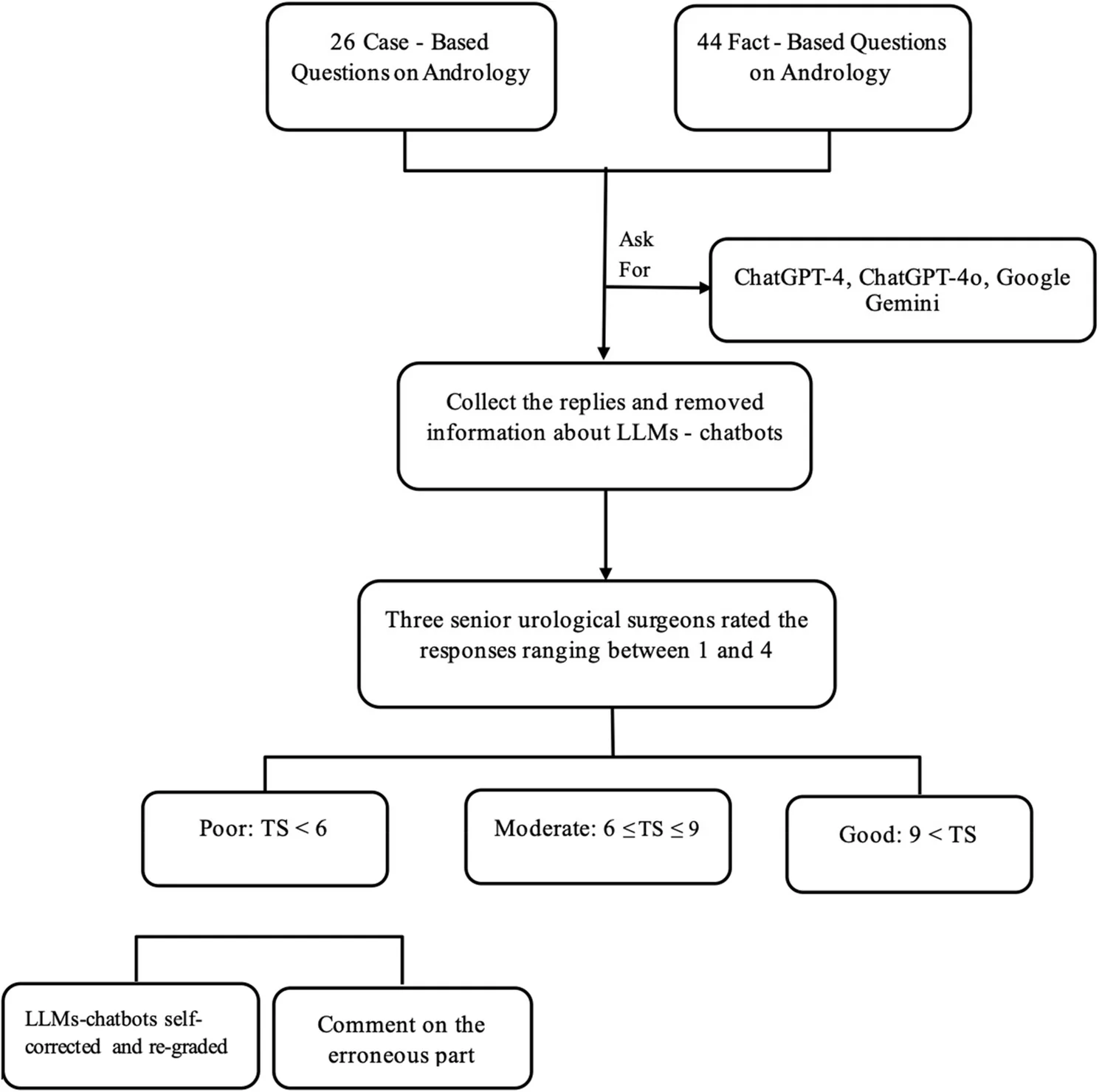
Flowchart of the overall study design

Three experienced urologists independently evaluated the responses using a scoring system ranging from 1 to 4, where 1 indicated a completely incorrect response, 2 indicated a partially correct response containing misinformation, 3 indicated a correct but insufficient response, and 4 indicated a correct and sufficient response. This scoring system was specifically developed for the present study to evaluate the accuracy, adequacy, and presence of misinformation in structured, guideline-based question-and-answer responses.

The consistency among evaluators in assessing the chatbot responses was analyzed using Fleiss’ kappa coefficient. The Fleiss’ kappa values were 0.690, 0.768, and 0.649, respectively, indicating substantial interrater agreement within our scoring system.

The responses were classified on the basis of the total score (TS): TS > 9 was categorized as “good”, 6 ≤ TS ≤ 9 was categorized as “moderate”, and TS < 6 was categorized as “poor”. Responses classified as “poor” were further analyzed by a urology expert, who identified and detailed any incorrect or misleading information for additional review. These responses were then self-corrected within the LLM chatbot system by using the following prompt: “This response does not seem accurate. Could you review it again?”.

The revised responses were then collected and converted into plain text format, with all chatbot-related identifiers removed and the sequence of responses altered. The corrected content was re-evaluated by three experienced urologists. This second evaluation was conducted one week after the initial scoring, and the evaluators were not informed that they were assessing self-corrected versions of the responses.

### Statistical analysis

The normality of the data distribution was assessed using the Kolmogorov–Smirnov and Shapiro–Wilk tests. One-way analysis of variance (ANOVA) was performed to compare differences among groups. For variables showing significant differences, Bonferroni-adjusted post hoc tests were applied for pairwise comparisons. Agreement among expert evaluations of chatbot responses was determined using Fleiss’ kappa coefficient and categorized as follows: 0–0.20, poor agreement; 0.21–0.40, fair agreement; 0.41–0.60, moderate agreement; 0.61–0.80, substantial agreement; and 0.81–1.00, almost perfect agreement. The self-correction capacity of the chatbots was analyzed using a paired-samples t-test by comparing precorrection and postcorrection scores. Effect size was assessed using Cohen’s d coefficient and reported with 95% confidence intervals. All the statistical analyses were conducted using SPSS (IBM SPSS Statistics, version 26.0), and the significance level was set at *p* < 0.05.

## Results

A total of 70 questions were evaluated in this study, consisting of 62.9% (*n* = 44) fact-based questions and 37.1% (*n* = 26) case-based questions. Among the andrology-related subgroups, the most frequently addressed categories were male hypogonadism (21.4%, *n* = 15), penile curvature (17.1%, *n* = 12), and male infertility (15.7%, *n* = 11). Additionally, the studies included priapism (14.3%, *n* = 10), penile size abnormalities and dysmorphophobia (12.9%, *n* = 9), erectile dysfunction (11.4%, *n* = 8), and ejaculatory disorders (7.1%, *n* = 5). The questions were categorized into three key domains: general knowledge (25.7%, *n* = 18), diagnosis (31.4%, *n* = 22), and treatment (42.9%, *n* = 30).

As shown in Table 1, significant differences were observed in the numbers of words and characters generated by the chatbots in response to the case-based questions (*p* < 0.001). Gemini produced the greatest number of words (300.88 ± 58.53) and characters (2126.38 ± 420.34), whereas ChatGPT-4o generated more content than ChatGPT-4 did (*p* < 0.001). For fact-based questions, Gemini and ChatGPT-4o produced more words and characters than ChatGPT-4 did (*p* < 0.001), but no significant difference was found between Gemini and ChatGPT-4o. When all the responses to both case-based and fact-based questions were analyzed, ChatGPT-4o consistently produced significantly more content than ChatGPT-4 did, whereas Gemini generated the highest output overall (*p* < 0.001).

**Table 1:** Word and character counts of responses provided by chatbots for case-based and fact-based questions

	ChatGPT-4	ChatGPT-4o	Google Gemini	p
Case-Based Questions				
Word Count	162.81±52.55^a^	201.46±49.87^b^	300.88±58.53^c^	<0.001*
Character Count	1169.96±363.99^a^	1556.77±426.44^b^	2126.38±420.34^c^	<0.001*
Fact-Based Questions				
Word Count	164.61±63.04^a^	231.52±75^b^	257.93±74.74^b^	<0.001*
Character Count	1199.18±449.60^a^	1757.52±644.76^b^	1825.98±544.47^b^	<0.001*
Total				
Word Count	163.94±58.97	220.36±67.98	273.89±71.83	<0.001*
Character Count	1188.33±417.34	1682.76±578.33	1937.56±519.74	<0.001*

Bold values indicate statistically significant p-values (*p* < 0.05)

^*^p<0,001, ^a,b,c:^ The difference between the means of different groups of letters is statistically significant

In the diagnosis category, ChatGPT-4o (11.22 ± 1.99) achieved a significantly higher total score than ChatGPT-4 (10.13 ± 2.33) and Gemini (9.95 ± 2.78) did (*p* = 0.040). As shown in Table 2, no significant difference was found among the chatbots in the general knowledge category. In the treatment category, ChatGPT-4o (10.53 ± 3.12) had a significantly higher score than ChatGPT-4 (8.36 ± 3.22) and Gemini (7.86 ± 3.66) did (*p* < 0.001).

**Table 2:** Total scores for chatbots based on question categories

Question Category	n	ChatGPT-4	ChatGPT-4o	Gemini	p
Diagnosis	22	10.13±2.33^a^	11.22±1.99^b^	9.95±2.78^a^	0.040*
General Information	18	7.77±3.70^a^	9.33±3.59^a^	8.27±3.35^a^	0.274
Treatment	30	8.36±3.22^a^	10.53±3.12^b^	7.86±3.66^a^	<0.001*

Bold values indicate statistically significant p-values (*p* < 0.05)

^*^p<0,05, ^a,b,c:^ The difference between the means of different groups of letters is statistically significant

As shown in Table 3, no significant differences were found among the three chatbots in the diagnosis and general information categories. However, a significant difference was observed in the treatment category (*p* < 0.001), with ChatGPT-4o achieving the highest accuracy, with 50% of its responses classified as good, 5.3% as moderate, and 19% as poor.

**Table 3:** Comparison of chatbot response accuracy classifications by category

	ChatGPT-4			ChatGPT-4o			Google Gemini			p
	Poor	Moderate	Good	Poor	Moderate	Good	Poor	Moderate	Good	
Diagnosis	1(20)^a^	6(60)^a^	15(29.40)^a^	1(20)^a^	1(10)^a^	20(39.2)^a^	3(60)^a^	3(30)^a^	16(31.4)^a^	0.188
General Information	6(42.9)^a^	4(30.8)^a^	8(29.60)^a^	4(28.6)^a^	3(23.1)^a^	11(40.7)^a^	4(28.6)^a^	6(46.2)^a^	8(29.6)^a^	0.693
Treatment	6(28.6)^a^	12(63.2)^a^	12 (24)^a^	4(19)^a^	1(5.3)^b^	25(50)^b^	11(52.4)^a^	6(31.6)^a. b^	13(26)^a^	**<0.001**

Bold values indicate statistically significant p-values (*p* < 0.05)

^*^p<0,05, ^a,b,c:^ The difference between the means of different groups of letters is statistically significant

When the impact of chatbot models on total scores was examined, the differences for both case-based (*p* = 0.009) and fact-based (*p* = 0.044) questions were statistically significant. For the case-based questions, ChatGPT-4o (M = 11.42, SD = 1.85) achieved the highest score, whereas Google Gemini (M = 9.27, SD = 3.24) had the lowest score. The score for ChatGPT-4o was significantly greater than that for Gemini, whereas no significant difference was detected between ChatGPT-4 and Gemini. As shown in Table 4, a similar pattern was observed for fact-based questions and the overall evaluation, in which ChatGPT-4o obtained the highest score, whereas the difference between ChatGPT-4 and Gemini was not statistically significant.

**Table 4:** Comparison of total scores for chatbot responses to case-based and fact-based questions

	ChatGPT-4	ChatGPT-4o	Google Gemini	p
Case-Based Questions				
Total Score	9.65±2.57^a^	11.42±1.85^b^	9.27±3.24^c^	0.009*
Fact-Based Questions				
Total Score	8.25±3.45^a^	9.86±3.40^b^	8.25±3.48^b^	0.044*
Total				
Total Score	8.77±3.21	10.44±3	8.63±3.41	0.001*

Bold values indicate statistically significant p-values (*p* < 0.05)

^*^p<0,05, ^a,b,c:^ The difference between the means of different groups of letters is statistically significant

In the analysis comparing the total scores of chatbot responses across andrology subgroups, a significant difference was found in the penile curvature subgroup (*p* = 0.003). According to the post hoc analysis, ChatGPT-4o (M = 11.33, SD = 2.30) achieved a significantly higher score than ChatGPT-4 (M = 7.75, SD = 3.10) and Google Gemini (M = 6.83, SD = 3.66). However, no statistically significant differences were observed among the chatbot models in the other andrology subgroups.

Paired-samples t tests assessing the self-correction capacity of the chatbots demonstrated a significant increase in postcorrection scores across all three models. As shown in Table 5, the initial numbers of poor responses were as follows: ChatGPT-4, 13 questions; ChatGPT-4o, 9 questions; and Google Gemini, 18 questions. Following self-correction, the scores improved significantly (*p* < 0.01). Analysis of Cohen’s d values revealed that ChatGPT-4o had the largest effect size (d = − 1.214), followed by ChatGPT-4 (d = − 0.954), whereas Google Gemini exhibited the weakest correction performance (d = − 0.738). These findings indicate that ChatGPT-4o demonstrated the strongest self-correction capability among the evaluated models. Representative examples illustrating changes in responses following corrective prompting across the evaluated large language models are presented in Supplementary Table 2.

**Table 5:** Self-Correction Capacity of ChatGPT-4, ChatGPT-4o, and Google Gemini

	TS		p	Cohen’s d Effect Size
	Initial	Self-correction		
ChatGPT-4	3.30±0.48	4.84±1.86	0.005*	-0.954 (95% CI:-1.602 -0.279)
ChatGPT-4o	3.37±0.51	7.37±3.02	0.011*	-1.214 (95% CI:-2.123 -0.259)
Google Gemini	3.66±0.76	6.11±3.21	0.006*	-0.738 (95% CI: -1.253 -0.206)

Bold values indicate statistically significant p-values (*p* < 0.05)

^*^p<0,05, indicates significant difference

## Discussion

Large language models and NLP tools are designed to comprehend natural language and generate responses to text-based prompts. These AI-driven chatbots generate responses on the basis of a vast corpus of publicly available text data, including academic articles, websites, and other textual sources [9]. Generative AI holds significant potential for medical applications, including enhancing diagnosis, image analysis, personalized treatment, and patient monitoring, thereby improving the efficiency and effectiveness of healthcare services [10, 11].

Although LLMs can mimic human reasoning, concerns regarding their reliability in healthcare applications persist. They demonstrate significant potential for medical applications, including literature reviews, medical education, and patient data processing [12, 13].

To the best of our knowledge, this is the first study to compare the accuracy, consistency, and self-correction capacity of responses generated by LLMs to andrology-related questions within the context of the current literature and recent research.

The number of studies evaluating the performance of artificial intelligence chatbots across different medical specialties has increased considerably in recent years. Recent studies in the fields of urodynamics, bladder cancer, and apheresis have reported significant differences among large language models in terms of information quality, reliability, accuracy, and readability [14–16]. These findings suggest that the performance of AI-generated medical information may vary depending on the model used. Similarly, in the present study, performance differences were observed among the models in their responses to guideline-based questions in andrology.

The literature suggests that chatbots may vary in response length. A comparative study of medical inquiries demonstrated that ChatGPT provided more accurate but shorter responses than Gemini did [17]. In contrast, another study evaluating AI-generated brochures for emergency medical conditions reported that, compared with Gemini, ChatGPT produced responses with a higher word count and more sentences [18]. In our study, Google Gemini generated the longest responses, whereas ChatGPT-4o provided balanced and moderately long responses. However, response length does not necessarily ensure accuracy or clinical relevance. These findings highlight that not only comprehensiveness but also accuracy, clarity, and concordance with clinical guidelines are crucial considerations in AI-assisted medical information delivery. Therefore, the findings of the present study primarily reflect guideline adherence and factual consistency rather than a direct assessment of clinically meaningful reasoning. Consequently, high performance scores should not be interpreted as evidence that the models are capable of performing complex clinical reasoning or making decisions under uncertainty in real-world clinical scenarios.

Studies evaluating the performance of LLMs in medicine highlight both their potential and limitations in clinical decision-support processes. A study on contraceptive methods revealed that ChatGPT-4o had the lowest incidence of misinformation, whereas Gemini demonstrated superior response relevance [19]. In an analysis of pediatric nephrology inquiries, ChatGPT-4o initially provided more accurate responses, whereas Gemini 1.5 showed greater improvement over time [20]. In the management of urolithiasis, ChatGPT-4o provided the most guideline-adherent recommendations on the basis of the EAU guidelines, whereas Gemini demonstrated the lowest performance [21]. In our study, when the total scores of the chatbots were examined across different question categories, ChatGPT-4o achieved significantly higher scores than ChatGPT-4 and Google Gemini did in both the diagnosis and treatment categories. These findings suggest that ChatGPT-4o demonstrates more consistent performance than the other models do in terms of response to guideline-based questions related to andrology. However, as highlighted in previous studies, AI-based models are not yet sufficient for making independent medical decisions and should be used under expert supervision.

When comparing the diagnostic performance of LLMs in medicine, ChatGPT-4o was found to be significantly superior to Gemini Advanced for retinal diseases, with an accuracy rate of 62% [22]. Similarly, ChatGPT-4o was reported to outperform Gemini in the initial assessment of neurogenetic disorders [23]. However, studies have also shown that all models exhibit certain deficiencies in diagnostic accuracy and may provide inappropriate information. In the diagnosis of osteoarthritis, ChatGPT-4o outperformed the other models by achieving the highest accuracy rate, with a sensitivity of 92.3% [24]. A similar trend was observed in our study. Although no significant difference in accuracy was observed among the models in the diagnosis category, ChatGPT-4o provided more structured responses with greater concordance with clinical guidelines.

In a study evaluating the accuracy of chatbots for regenerative endodontic treatment, responses to 23 questions were analyzed, and Gemini had the lowest performance, with an accuracy rate of 48% [25]. Similarly, in a study analyzing glaucoma surgical cases, ChatGPT-4 exhibited superior performance in analysis and recommendations compared with Gemini [26]. In our study, ChatGPT-4o achieved the highest accuracy score in the treatment category, whereas Google Gemini demonstrated a lower level of accuracy. Notably, in the treatment domain, ChatGPT-4o presented guideline-based information in a more consistent and structured manner. While our findings suggest that AI-based language models may play a supportive role in the delivery of guideline-oriented medical information, they also underscore the need to account for variability in accuracy when considering clinical applications. The performance of chatbots may be influenced by their ability to present guideline-based information in a systematic and consistent manner.

In the literature, a study evaluating 25 clinical andrology cases reported that specialist physicians achieved the best performance, while ChatGPT-4 outperformed residents and other chatbots. Although ChatGPT-3.5 performed better than Gemini did, it did not reach the performance level of ChatGPT-4 or specialist physicians [27]. In a study testing the reliability of large language models in the management of advanced head and neck malignancies, ChatGPT-4 generally outperformed Gemini Advanced in terms of guideline adherence and comprehensive treatment planning [28]. In our study, the total scores for the ChatGPT-4o responses to case-based and fact-based questions were significantly higher than those for the ChatGPT-4 and Google Gemini responses. While fact-based questions primarily assess the retrieval of guideline-based knowledge, case-based questions require interpretation of clinical information within a specific scenario and therefore involve a relatively greater degree of clinical reasoning. This finding suggests that ChatGPT-4o may demonstrate more consistent performance both in presenting guideline-based information and in interpreting structured clinical scenarios. Nevertheless, both question types were evaluated within a predefined guideline framework and do not fully reflect real-world clinical decision-making processes or complex clinical situations involving uncertainty.

There are a limited number of studies examining the accuracy of responses generated by AI models to medical questions and their self-correction capabilities. In a study comparing the performance of large language models in glucocorticoid-induced osteoporosis, the self-correction ability of ChatGPT-3.5 and ChatGPT-4 was better than that of Gemini, whereas Gemini did not significantly improve during the correction process [29]. Similarly, Su Z et al. compared the performance of large language models in providing information on cataract care, with ChatGPT-4o performing best in terms of providing accurate and comprehensive responses, showing improvement during the self-correction process, and effectively analyzing its errors [30]. The findings of our study are consistent with those of previous research, which demonstrated that ChatGPT-4o had the highest self-correction capacity, whereas Google Gemini had the lowest self-correction capacity. This may suggest that ChatGPT-based models respond more effectively to corrective prompts and are able to improve their responses when additional guidance is provided. However, because the self-correction process was triggered by explicitly informing the models that their responses might be inaccurate, these findings may reflect prompt sensitivity and responsiveness to feedback rather than true autonomous error awareness. Therefore, self-correction performance should be interpreted with caution, and the observed results should not be considered a direct indicator of an independent ability to recognize errors. To improve the reliability of LLMs in medical consultation, their error analysis capabilities should be further enhanced. Large language models continue to evolve rapidly, and their potential roles in clinical practice are being increasingly explored. Although the integration of artificial intelligence into urological practice offers important opportunities, the safe and effective use of these technologies requires expert oversight and continuous feedback.

The primary contribution of the present study is not merely the demonstration of performance differences among various models. Numerous studies have reported that more recent large language models can achieve higher performance than earlier-generation models in guideline-based question-answering tasks. However, the current study provides additional value to the literature by focusing specifically on andrology; evaluating diagnostic, treatment-related, factual, and case-based questions as separate categories; and comparing the self-correction capacities of different models. This approach enables the evaluation not only of the guideline concordance of responses generated by large language models but also of their responsiveness to feedback and their potential for self-correction. From a clinical perspective, these findings provide additional insight into the domains in which AI-assisted systems may demonstrate more consistent performance and further expand the existing evidence supporting their use as supportive tools under expert supervision.

Even when guideline-based frameworks are used, excessive reliance on large language models for generating clinical responses may entail potential risks, including the generation of inaccurate or contextually inappropriate information, reliance on outdated guideline content, and insufficient consideration of individual patient characteristics. Therefore, AI-based systems should be regarded as supportive tools and used under expert supervision in clinical practice.

This study has several limitations. First, it was restricted to specific clinical scenarios and was conducted exclusively in English. The predominantly guideline-based, factual, and fully specified nature of the questions limits the assessment of complex clinical reasoning processes required in situations involving uncertainty, incomplete data, or conflicting clinical information. This may have resulted in an overestimation of large language model performance compared with real-world clinical practice. The idealized structure of the case scenarios further limits the direct generalizability of the findings to routine clinical settings. Furthermore, the self-correction prompt applied to responses classified as poor represents a standardized and artificial approach and may not fully reflect the dynamic nature of physician–AI interactions in real clinical environments. Similarly, each question was evaluated as an independent session, and the multistep, iterative user–AI interactions commonly encountered in real-world clinical practice were not taken into account. In addition, the 1–4 scoring system used in this study was specifically developed for this purpose and has not been validated in independent cohorts or by external evaluators. Finally, the rapid and continuous evolution of large language models, the evaluation of only a limited number of systems, and the exclusion of other widely used models, such as Claude, limit the temporal validity and generalizability of the findings. Therefore, the present results reflect only the specific model versions evaluated in this study and may not be directly applicable to future versions.

## Conclusion

Among the models evaluated in this study, ChatGPT-4o achieved higher scores than the other models did in terms of the consistency of its responses to guideline-based andrology questions and self-correction performance. However, because the present study did not assess real-world clinical reasoning or safety-critical decision-making processes, the findings should be considered exploratory in nature. Accordingly, these results suggest that AI-assisted systems should be regarded as supportive tools under expert supervision rather than as independent decision-makers in clinical practice. Future improvements in responsiveness to feedback, error analysis capabilities, and self-correction mechanisms may further enhance the reliability of AI-based medical consultation systems.

## Supplementary Information

Supplementary Material 1.

Supplementary Material 2.

## Data Availability

The datasets generated and/or analyzed during the current study are available from the corresponding author upon reasonable request. No individual patient-level data were used in this study.
