# Supplementary material for: The doctors of the future: the competition of ChatGPT-4, ChatGPT-4 omni, and Gemini 2.0 Flash in andrology
Source: BMC Urol. 2026 Jul 7;26:231. doi: 10.1186/s12894-026-02248-5 (PMC13625468; doi:10.1186/s12894-026-02248-5)
Supplement: Supplementary file 1 [file 12894_2026_2248_MOESM1_ESM.docx]

**Supplementary Table-1 :** **Andrology-Related Questions Based on the European Association of Urology (EAU) 2024 Guideline Recommendations**

| **Question number** | **Andrology subspeciality** | **Question** |
| --- | --- | --- |
| 1 | Male Hypogonadism | A 40-year-old male patient presents with progressively worsening fatigue, loss of libido, and erectile dysfunction (ED) over the past five months. His physical examination is unremarkable. Laboratory findings reveal total testosterone level of 5.5 nmol/L, elevated serum prolactin level, and deficiencies in basal anterior pituitary hormones. What should be the next step in the diagnostic approach for this patient? |
| 2 | Male Hypogonadism | What are the relative contraindications for testosterone therapy in hypogonadism? |
| 3 | Male Hypogonadism | What is the threshold total testosterone level (in nmol/L) for initiating testosterone therapy in the presence of hypogonadal symptoms? |
| 4 | Male Hypogonadism | In which patient groups should late-onset hypogonadism screening be performed? |
| 5 | Male Hypogonadism | A 32-year-old male patient presents to the outpatient clinic with complaints of low sexual desire and fatigue. His physical examination is unremarkable, and he has no additional medical conditions in his history. Laboratory tests reveal low total testosterone level. The urologist suspects secondary hypogonadism. Which laboratory test should be requested to further evaluate this diagnosis? |
| 6 | Male Hypogonadism | Which biochemical and hormonal analyses should be performed to differentiate between primary and secondary forms of hypogonadism? |
| 7 | Male Hypogonadism | Given the low specificity of structured interviews and questionnaires used for the diagnosis of late-onset hypogonadism, what role in screening protocols should they have? |
| 8 | Male Hypogonadism | A 38-year-old male patient presents to the outpatient clinic with complaints of low energy, fatigue, and decreased sexual desire. The physician decides to evaluate total testosterone levels. What factors should the physician consider to enhance measurement accuracy and minimize biological variability? |
| 9 | Male Hypogonadism | Should testosterone therapy be used in eugonadal men? |
| 10 | Male Hypogonadism | A 43-year-old male patient with no known medical conditions or medication use presents with mild ED, low energy, and fatigue for the past six months. His physical examination is unremarkable, and his morning total testosterone level is 220 ng/dL, with other hormone levels within the normal range. What is the first-line treatment option for erectile dysfunction and hypogonadism in this patient? |
| 11 | Male Hypogonadism | Is testosterone therapy recommended to improve cognitive function, physical performance, and quality of life in elderly men? |
| 12 | Male Hypogonadism | What assessments should be performed before initiating testosterone therapy in a patient? |
| 13 | Male Hypogonadism | Which treatment can be used to restore fertility in men with secondary hypogonadism? |
| 14 | Male Hypogonadism | How should PSA levels be monitored in a patient with a history of prostate cancer who is receiving testosterone therapy? |
| 15 | Male Hypogonadism | Which biochemical parameters should be monitored during testosterone therapy? |
| 16 | Erectile Dysfunction | How should the initial evaluation be conducted in a male patient presenting with ED, and what approach should be taken to identify potential underlying causes? |
| 17 | Erectile Dysfunction | Which tools or psychometric questionnaires should be used to assess the severity of erectile dysfunction? |
| 18 | Erectile Dysfunction | Which PDE5 inhibitor used in the treatment of erectile dysfunction is associated with myalgia and back pain as side effects? |
| 19 | Erectile Dysfunction | Is there a difference in efficacy and safety among the various penile implants or surgical approaches used in the treatment of ED? |
| 20 | Erectile Dysfunction | Which psychological approaches can be effective in overcoming ED in treatment processes that involve partner participation? |
| 21 | Erectile Dysfunction | Is there a role for ESWT in the treatment of erectile dysfunction? |
| 22 | Erectile Dysfunction | Does penile rehabilitation with PDE5 inhibitors after radical prostatectomy increase the likelihood of spontaneous erectile function recovery? |
| 23 | Erectile Dysfunction | A 45-year-old male patient presents with a six-month history of ED. His medical history includes hypertension, obesity, and smoking. Laboratory findings reveal a fasting blood glucose level of 110 mg/dL, HbA1c of 6.2%, and a total testosterone level of 280 ng/dL. The patient reports a reduction in morning erections and difficulty maintaining an erection during sexual activity. What are the current treatment approaches for managing this patient? |
| 24 | Ejaculation Disorders | Are routine laboratory or physiological tests performed for the evaluation of premature ejaculation? |
| 25 | Ejaculation Disorders | A 50-year-old male patient presents with a four-month history of ED, premature ejaculation, perineal pain, dysuria, and frequent urination. His medical history includes diabetes and hypertension. Physical examination reveals perineal tenderness. Laboratory findings: Total testosterone: 320 ng/dL, PSA: 1.2 ng/mL, Urinalysis: Leukocytes (+), Bacteria (+), TRUS: Prostate volume of 35 cc with inflammatory changes observed. What is the primary concern for this patient, and what recommended treatment approach should be used? |
| 26 | Ejaculation Disorders | In a case where premature ejaculation and erectile dysfunction coexist, which condition should be prioritized in the initial treatment plan? |
| 27 | Ejaculation Disorders | Can PDE5 inhibitors be used as a standalone treatment in a male patient with premature ejaculation but without ED? |
| 28 | Ejaculation Disorders | A 29-year-old male patient presents with lifelong premature ejaculation that began after marriage. He reports that ejaculation occurs within the first few minutes of sexual intercourse, causing distress for both himself and his partner. His general health history is unremarkable, with no complaints of erectile dysfunction or decreased libido. Physical examination and laboratory tests are normal. What are the first-line treatment options for premature ejaculation in this patient? |
| 29 | Penile Curvature | A 17-year-old male patient presents to the urology clinic with his family, reporting a complaint of penile curvature that he has noticed for several years. He describes a downward curvature of the penis during erection, which causes him esthetic concerns. The patient has not yet been sexually active and is concerned about whether this condition will negatively affect his future sexual life. On physical examination, the penis appears normal in the flaccid state. However, the patient is asked to provide erection photographs, which reveal a significant ventral curvature of approximately 40 degrees. No palpable plaques or indurations are detected on examination. What is the recommended treatment approach and optimal timing for intervention in this patient? |
| 30 | Penile Curvature | What aspects should be assessed during physical examination to support the diagnosis of Peyronie disease (PD) and determine the disease progression? |
| 31 | Penile Curvature | Which treatment methods have been proven ineffective or are not recommended in the management of PD? |
| 32 | Penile Curvature | A 41-year-old male patient presents to the clinic with complaints of penile curvature and difficulty during sexual intercourse, which he has noticed over the past year. History reveals that the curvature has been stable for the last six months, and the patient reports no pain. Physical examination identifies a dorsal penile curvature of approximately 45°, with palpable Peyronie plaques. The patient does not wish to undergo surgical treatment but desires correction of the curvature. What is the most appropriate treatment option for this patient? |
| 33 | Penile Curvature | A 52-year-old male patient presents with complaints of penile curvature and pain during sexual intercourse. He reports that his symptoms began approximately three months ago and have progressively worsened over time. Physical examination reveals a dorsal plaque and a penile curvature of approximately 35°. The patient has no known chronic medical conditions. Should intralesional steroid therapy be recommended for pain relief and curvature correction in this patient? |
| 34 | Penile Curvature | How should the management of penile pain be planned in the acute phase of PD? |
| 35 | Penile Curvature | Outside the scope of clinical research, can intralesional platelet-rich plasma or hyaluronic acid be used alone or in combination with oral therapy to reduce penile curvature, plaque size, or pain? |
| 36 | Penile Curvature | A 53-year-old male patient presents with an 8-month history of penile curvature and difficulty during sexual intercourse. He reports that his symptoms were initially painful, but the pain has resolved over the past 4 months. Physical examination reveals a dorsal penile curvature of 40° with palpable penile plaques. The patient states that he does not wish to undergo surgical intervention. Should ESWT be recommended for this patient? |
| 37 | Penile Curvature | A 47-year-old male patient presents with a one-year history of penile curvature and difficulty during sexual intercourse. He reports that the curvature has been stable for the past six months. Physical examination reveals a dorsal penile curvature of 50° with palpable plaques. The patient experiences a sensation of penile shortening and reduced erectile strength but responds well to PDE5 inhibitors. He is interested in surgical treatment options and expresses a desire for both curvature correction and improvement in sexual function. Which factors should be considered preoperatively in this patient? |
| 38 | Penile Curvature | Why is the sliding technique not recommended in the surgical treatment of PD? |
| 39 | Penile Curvature | Are synthetic grafts a safe and effective option in the surgical treatment of PD? |
| 40 | Penile Curvature | A 55-year-old male patient presents with Peyronie disease and worsening ED. He reports that he has not responded to pharmacotherapy, including PDE5 inhibitors, and is unable to achieve a satisfactory erection. Physical examination reveals a dorsal plaque and 40° dorsal penile curvature. Surgical treatment is considered to improve his quality of life. What is the most appropriate surgical treatment option for this patient? |
| 41 | Penile Size Abnormalities and Dysmorphophobia | Which condition or disorder should be considered in patients who have a normally sized penis but perceive it as shorter than normal? |
| 42 | Penile Size Abnormalities and Dysmorphophobia | A 60-year-old male patient with a BMI of 34 presents with complaints of difficulty urinating, sexual discomfort, and dissatisfaction with the appearance of his penis. He has a history of lichen sclerosus. Physical examination reveals prominent suprapubic fat, a buried penis, and only partial visibility of the glans. The patient states that his symptoms negatively impact his daily life and self-confidence. What primary diagnosis should be considered in this patient? |
| 43 | Penile Size Abnormalities and Dysmorphophobia | In which cases should the necessity for psychotherapy be evaluated before surgical or invasive penile enlargement procedures? |
| 44 | Penile Size Abnormalities and Dysmorphophobia | A 7-year-old male child is brought in by his family with concerns about small penile size. Physical examination reveals that the penile length is below the normal range for his age. History indicates no significant genital anomalies since birth, but growth and development have been reported as below age expectations. Laboratory evaluation shows low testosterone levels, while gonadotropin levels are appropriate for age. A consultation with the pediatric endocrinology department has been requested. What is the most appropriate treatment option to normalize penile size in this child? |
| 45 | Penile Size Abnormalities and Dysmorphophobia | A 22-year-old male patient presents with concerns about having small penile size. He reports completing puberty normally and states that he has never been diagnosed with any hormonal disorder. Physical examination and measurements confirm that his penile size is within the normal range, and hormonal test results are also normal. The patient requests hormonal therapy to increase penile size. Which treatment option should the urologist avoid recommending in this case? |
| 46 | Penile Size Abnormalities and Dysmorphophobia | A 56-year-old male patient with a BMI of 36 presents with complaints of buried penis, difficulty during sexual intercourse, and recurrent urinary tract infections. Physical examination reveals prominent suprapubic fat, a completely buried penis, and scar tissue. The patient is diagnosed with acquired buried penis in adulthood, and surgical treatment is planned. What are the primary approaches to optimize surgical outcomes and minimize complications in this patient? |
| 47 | Penile Size Abnormalities and Dysmorphophobia | A 34-year-old male patient presents with concerns about inadequate penile size. Physical examination and measurements confirm that his penile size is within the normal range. The patient states that he has been researching penile lengthening options and specifically requests information about surgical procedures. Should penile prosthesis implantation, penile disassembly (demontage), or sliding techniques for lengthening be recommended for this patient? |
| 48 | Penile Size Abnormalities and Dysmorphophobia | Can bioresorbable scaffolds and the subcutaneous penile implant (Penuma®) be used to increase penile girth? |
| 49 | Penile Size Abnormalities and Dysmorphophobia | How appropriate is the use of grafts for penile girth enhancement for cosmetic or reconstructive purposes? |
| 50 | Priapism | A 19-year-old male patient is brought to the emergency department by his family with complaint of painful penile erection lasting for the past 8 hours. The patient reports having experienced similar but shorter episodes in the past. His medical history reveals a diagnosis of sickle cell anemia, for which he receives regular treatment, including hydroxyurea. On physical examination, the corpora cavernosa are firm, while the glans penis remains soft. Abdominal examination is unremarkable. Which physical examination findings and laboratory tests should be evaluated to confirm the diagnosis of priapism and determine its subtype? |
| 51 | Priapism | In which cases should hemoglobinopathy screening be performed in patients with low-flow priapism? |
| 52 | Priapism | Is the analysis of blood gas parameters from aspirated penile blood important for distinguishing between ischemic and non-ischemic priapism? |
| 53 | Priapism | A 28-year-old male patient presents to the emergency department with a painful penile erection lasting for more than 7 hours. His medical history reveals a diagnosis of sickle cell anemia. A urology consultation is requested. On physical examination, the corpora cavernosa are firm, while the glans penis remains soft. The patient reports severe pain. What should the first step in the management of this patient be? |
| 54 | Priapism | In priapism induced by intracavernosal injection of vasoactive agents, should corpus cavernosum aspiration be the first step for management? |
| 55 | Priapism | A 44-year-old male patient presents to the emergency department with a complaint of painful, persistent erection that started approximately 8 hours ago. He has no known systemic diseases or regular medication use. On physical examination, rigidity of the penile shaft is noted, and cavernous blood gas analysis reveals venous characteristics, confirming ischemic priapism. The patient undergoes corporal aspiration and intracavernosal phenylephrine injection. However, upon reevaluation 35 minutes later, the erection persists. What is the most appropriate next treatment in this case? |
| 56 | Priapism | A 30-year-old male patient with a history of sickle cell anemia presents to the emergency department with ischemic priapism lasting 48 hours. Initial management includes aspiration and intracavernosal phenylephrine injection, but these measures fail. The patient subsequently undergoes a distal shunt procedure. After surgery, he is discharged in stable condition. At his 3-month follow-up, the patient reports ED and requests penile prosthesis implantation. What complications have increased risk if penile prosthesis implantation is performed in the early postoperative period? |
| 57 | Priapism | What treatment approaches should be implemented for recurrent (stuttering) priapism? |
| 58 | Priapism | Which imaging modality should be used prior to planning embolization therapy for non-ischemic priapism? |
| 59 | Priapism | What should the next step for treatment of non-ischemic priapism be if conservative therapies fail? |
| 60 | Male Infertility | How should the evaluation and management of a 34-year-old male patient presenting to the urology clinic with a 3-year history of infertility be planned? |
| 61 | Male Infertility | In which cases should sperm DNA fragmentation testing be performed for the evaluation of male infertility? |
| 62 | Male Infertility | Should hormonal evaluation be performed in all cases of oligozoospermia and azoospermia? |
| 63 | Male Infertility | A 27-year-old male patient presents with a two-year history of infertility despite unprotected sexual intercourse. Semen analysis reveals a sperm concentration of 3 million/mL with severely impaired motility and morphology. There is no history of testicular trauma, surgery, or family history of genetic disorders. On physical examination, testicular size is normal, and no varicocele is detected. Laboratory tests show mildly elevated FSH and LH levels, while testosterone remains within the normal range. Should karyotype analysis and genetic counseling be recommended for diagnostic purposes in this patient? |
| 64 | Male Infertility | Should Y chromosome microdeletion testing be performed in men with a sperm concentration of ≤1 million sperm/mL? |
| 65 | Male Infertility | Can testicular sperm extraction be performed in patients with complete deletions of the AZFa and AZFb regions in male infertility? |
| 66 | Male Infertility | A 36-year-old male patient presents to the clinic for infertility evaluation after two years of regular, unprotected sexual intercourse without achieving pregnancy. Semen analysis reveals azoospermia. Physical examination shows normal testicular size and consistency, with epididymis appearing prominently full. Bilateral vas deferens are not palpable. Color Doppler ultrasonography confirms normal testicular vascularity, and renal ultrasonography reports no anomalies. Which genetic tests should be performed to clarify the diagnosis for this patient? |
| 67 | Male Infertility | A 37-year-old male patient presents to the clinic with a complaint of infertility despite three years of regular, unprotected sexual intercourse with his wife. Medical history: No significant illnesses or prior surgeries. Sexual history: Reports noticing a decrease in ejaculate volume. Semen analysis findings: Sperm concentration: 2 million/mL, Mild motility and morphology abnormalities. Ejaculate volume: 1.0 mL, pH: 6.8, Fructose: Negative, Physical examination: Normal testicular size, no palpable abnormalities, fullness and tenderness in the epididymis. Which diagnostic tests should be ordered to clarify the diagnosis in this case? |
| 68 | Male Infertility | In an adult patient evaluated for infertility in the urology clinic, an undescended testis is detected, and surgical intervention is planned. Which concurrent procedure is recommended for the diagnosis of intratubular germ cell neoplasia in situ ? |
| 69 | Male Infertility | Can fine-needle aspiration mapping and TESA be performed in patients with non-obstructive azoospermia ? |
| 70 | Male Infertility | A 16-year-old male patient is referred to the urology clinic during routine pediatric examination due to a significant size discrepancy between the right and left testicles. The patient has no complaints. Physical examination reveals prominent venous structures in the left scrotum. Scrotal ultrasound findings: Right testicular volume: 18 mL, Left testicular volume: 14 mL. Follow-up evaluation after six months confirms persistent testicular volume asymmetry. Is there an indication for varicocele surgery in this patient? |

*BMI: Body Mass Index, DNA: Deoxyribonucleic Acid, ED: Erectile Dysfunction, ESWT: Extracorporeal Shock Wave Therapy, FSH: Follicle-Stimulating Hormone, HbA1c: Glycated Hemoglobin A1c, LH: Luteinizing Hormone, PD: Peyronie’s Disease, PDE5i: Phosphodiesterase Type 5 Inhibitor, PSA: Prostate-Specific Antigen, TESA: Testicular Sperm Aspiration, TRUS: Transrectal Ultrasound,*
