# Supplementary material for: The doctors of the future: the competition of ChatGPT-4, ChatGPT-4 omni, and Gemini 2.0 Flash in andrology
Source: BMC Urol. 2026 Jul 7;26:231. doi: 10.1186/s12894-026-02248-5 (PMC13625468; doi:10.1186/s12894-026-02248-5)
Supplement: Supplementary file 2 [file 12894_2026_2248_MOESM2_ESM.docx]

**Supplementary Table 2. Representative Examples of Initial and Corrected Responses Following Self-Correction Across the Evaluated Large Language Models**

| **Question** | **Model** | **Initial Response** | **Corrected Response** | **Classification Change** |
| --- | --- | --- | --- | --- |
| Q54 Priapism | ChatGPT-4 | Yes. In priapism induced by intracavernosal injection of vasoactive agents, corporal aspiration may be considered as an initial treatment option. The aim of aspiration is to evacuate blood from the corpora cavernosa and thereby achieve detumescence. If aspiration is unsuccessful, intracavernosal sympathomimetic agents may be administered. Observation may also be considered depending on the patient’s clinical condition and response to treatment. | Corporal aspiration is an important therapeutic option in the management of priapism induced by intracavernosal injection therapy and is often considered among the initial treatment approaches. If an adequate response is not achieved with aspiration alone, intracavernosal alpha-adrenergic agents such as phenylephrine may be administered. Treatment should be individualized according to the duration of symptoms and the patient’s clinical presentation. | Poor→Poor |
| Q54 Priapism | ChatGPT-4o | Yes, corporal aspiration is one of the initial treatment options that may be used in the management of priapism induced by intracavernosal injection of vasoactive agents. Aspiration can facilitate detumescence by evacuating blood from the corpora cavernosa. If an adequate response is not achieved, sympathomimetic agents such as intracavernosal phenylephrine may be administered. The treatment approach should be tailored to the patient’s clinical condition and response to therapy. | No. In priapism induced by intracavernosal injection of vasoactive agents, corporal aspiration is not recommended as the first-line treatment. According to the EAU Guidelines, the preferred initial management is intracavernosal administration of a sympathomimetic agent, particularly phenylephrine. Corporal aspiration and irrigation may be considered in cases that do not respond adequately to phenylephrine therapy. Therefore, in injection-induced priapism, corporal aspiration is not the initial step but rather a subsequent treatment option that may be considered following failure of phenylephrine treatment. | Poor→Strong |
| Q54 Priapism | Gemini 2.0 Flash | Corporal aspiration is one of the initial treatment options for priapism induced by intracavernosal injection of vasoactive agents. By evacuating blood from the corpora cavernosa, aspiration may reduce intracavernosal pressure and facilitate detumescence. If this approach is unsuccessful, sympathomimetic agents such as intracavernosal phenylephrine may be administered. The treatment strategy should be determined based on the duration of symptoms, severity of pain, and the patient’s overall clinical condition. In some cases, additional interventional procedures may be required. | Upon reviewing my previous response, I would note that corporal aspiration represents an important initial treatment option in the management of priapism induced by intracavernosal injection of vasoactive agents. By evacuating blood from the corporal bodies, aspiration may facilitate detumescence and help relieve the prolonged erection. If aspiration alone is unsuccessful or an inadequate response is achieved, sympathomimetic agents such as intracavernosal phenylephrine may subsequently be administered. The choice of treatment should be guided by the duration of symptoms, clinical findings, and the patient’s response to therapy. In cases refractory to these measures, more advanced interventional approaches may be considered. | Poor→Poor |
| Q69 Male Infertility | ChatGPT-4 | Yes. In patients with non-obstructive azoospermia, both fine-needle aspiration mapping (FNAM) and testicular sperm aspiration (TESA) can be performed for the purpose of sperm retrieval. These techniques may assist in identifying areas of spermatogenesis within the testis and facilitate sperm retrieval. In selected patients, they may be considered as alternative approaches to other surgical sperm retrieval techniques. | In patients with non-obstructive azoospermia, both testicular sperm aspiration (TESA) and fine-needle aspiration mapping (FNAM) are techniques that can be utilized for the evaluation and retrieval of testicular sperm. These methods may be employed to investigate the presence of sperm within the testicular tissue. The choice of technique should be based on the patient’s clinical characteristics and the expertise and experience of the treating center. | Poor → Poor |
| Q69 Male Infertility | ChatGPT-4o | Yes. In patients with non-obstructive azoospermia, both fine-needle aspiration mapping (FNAM) and testicular sperm aspiration (TESA) can be performed for sperm retrieval. These techniques may help identify areas of active spermatogenesis within the testis and facilitate the retrieval of sperm. They may be considered among the available options, particularly in patients undergoing assisted reproductive techniques. | It should be noted that both fine-needle aspiration mapping (FNAM) and testicular sperm aspiration (TESA) can be performed in patients with non-obstructive azoospermia; however, their sperm retrieval success rates may be limited. Microdissection testicular sperm extraction (micro-TESE) is often preferred in this patient population because of its higher sperm retrieval rates. Nevertheless, TESA and FNAM may be considered alternative approaches in selected patients. | Poor → Moderate |
| Q69 Male Infertility | Gemini 2.0 Flash | Yes. In patients with non-obstructive azoospermia, both fine-needle aspiration mapping (FNAM) and testicular sperm aspiration (TESA) can be performed. Fine-needle aspiration mapping may help identify areas of active spermatogenesis within the testis, whereas TESA can be used for direct sperm retrieval. These techniques may be utilized in patients undergoing assisted reproductive procedures. The choice of method should be individualized based on the patient’s clinical characteristics and the experience of the treating center. | Fine-needle aspiration mapping (FNAM) and testicular sperm aspiration (TESA) are among the techniques that can be used for the evaluation of spermatogenesis and sperm retrieval in patients with non-obstructive azoospermia. FNAM may assist in identifying areas of active spermatogenesis within the testis, whereas TESA can be utilized to retrieve sperm from these regions. However, the success rates of these procedures may vary among patients, and the outcomes are largely dependent on the underlying testicular pathology. In addition, several other surgical sperm retrieval techniques are currently available. Therefore, the most appropriate approach should be determined based on the patient’s clinical characteristics, infertility history, and reproductive goals. | Poor → Poor |
